# Supplementary material for: MetaRibo-Seq measures translation in microbiomes
Source: Nat Commun. 2020 Jun 29;11:3268. doi: 10.1038/s41467-020-17081-z (PMC7324362; doi:10.1038/s41467-020-17081-z)
Supplement: Supplementary file 10 — Supplementary Data 7 [file 41467_2020_17081_MOESM10_ESM.zip › File2/Confidence_VeryHigh_Taxonomy/395341_out.krona.html]

Javascript must be enabled to view this page.

members
magnitude
magnitudeUnassigned
count
unassigned
taxon
rank

395341\_out

6

superkingdom
6
2

phylum
1224
6

1236
6
class

6
91347
order

543
6
family

561
5
genus

5
562

SRS012273\_contig\_number\_46582SRS017191\_contig\_number\_18775SRS017521\_contig\_number\_30268SRS049402\_contig\_number\_contig-100\_8.110434SRS144362\_contig\_number\_27174
species

547
1
genus

species

SRS1055050\_contig\_number\_contig-100\_1348.1349
1
539813
